# Interplay of Surface Charge and Pore Characteristics in the Immobilization of Lactate Oxidase on Bulk Nanoporous Gold Electrodes

**DOI:** 10.1021/acs.langmuir.4c04367

**Published:** 2025-02-20

**Authors:** Lara Marie Novak, Elisabeth Hengge, Eva-Maria Steyskal, Roland Würschum, Bernd Nidetzky

**Affiliations:** † Institute of Material Physics, Graz University of Technology, NAWI Graz, Petersgasse 16, 8010 Graz, Austria; ‡ Institute of Biotechnology and Biochemical Engineering, Graz University of Technology, NAWI Graz, Petersgasse 12, 8010 Graz, Austria

## Abstract

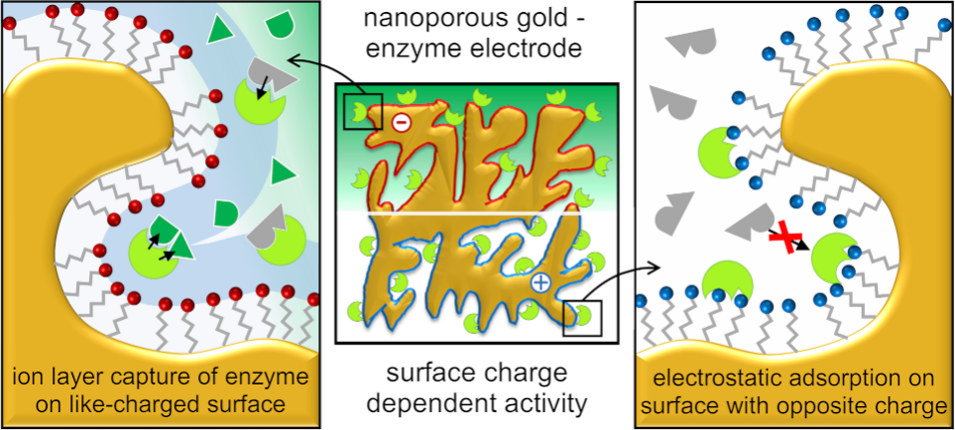

Immobilization of enzymes on (nano)porous metal carriers provides the foundation for an advanced design of bioelectrodes suitable for catalysis and sensing. However, interactions upon adsorption are still poorly understood, and so the efficient coupling of the enzymes to the electrode surface remains one of the major challenges. Here, we present a comprehensive study of the immobilization behavior of *Aerococcus viridans*l-lactate oxidase (LOx) on nanoporous gold (npAu) in dependence of electrode modification with differently charged self-assembled monolayers (SAMs). The highest activity (up to 14 U/g) and electrocatalytic response (sensitivity of 3.9 μA mM^–1^) were observed for a sulfonate-terminated SAM. This is contrary to enzyme behavior on conventional polymer carriers, and thus, the effect is specific to the metal electrodes. We propose the capture of the negatively charged LOx in a dense counterion layer in close proximity to the strongly negatively charged gold surface. Adsorption on positively charged amine-terminated SAMs resulted in a similar immobilization yield but gave much lower activity (4-fold). Importantly, the effect of the sulfonate SAM was strongly dependent on the npAu electrode pore size: the highest LOx activity (in U/cm^2^) was found with pores (diameter of ∼170 nm) supposedly large enough to facilitate enzyme diffusion into the porous structure during immobilization. Electrochemical sensing of H_2_O_2_ produced by the LOx reaction showed a 2.5-fold higher sensitivity for l-lactate on the negatively charged surface. Lixiviation studies supported the proposed layer capture and revealed a faster decline in the electrode activity with sulfonate surface modification. Collectively, the present study reveals enhanced activity of LOx on sulfonate-charged gold surfaces and a strong pore size dependence. These findings deepen the understanding of the immobilization behavior of LOx on charged nanoporous metals and have importance for the advanced design of enzyme electrodes.

## 1 Introduction

An understanding of the immobilization of enzymes on electron-conducting metal carriers provides the foundation for an advanced design of bioelectrodes.^1,2^ The bioelectrodes exploit the superior activity and selectivity of enzymes for catalysis and sensing.^3,4^ They find applications in environmental monitoring and healthcare, among others.^5,6^ Nanoporous gold (npAu) is a particularly promising material for use in these electrodes due to its unique properties.^7−10^ By electrochemical dealloying from an Au–Ag master alloy, a bicontinuous, self-standing porous structure is formed,^11^ exhibiting good structural stability, tunable pore size, and high electron transfer rate.^12−14^ The large surface-to-volume ratio and richness in defects result in a high intrinsic catalytic activity that enables enhanced electrochemical responses.^13,15^ Nevertheless, the coupling with enzymes is challenging as the bare metal surface is often associated with denaturation and provides only low immobilization yields.^16,17^ To improve the enzyme activity upon immobilization, modification of gold surfaces with self-assembled monolayers (SAMs) is commonly used.^18,19^ The molecules form ordered monolayers through a self-assembly process and are anchored to the surface via a covalent thiol–gold bond. The functional groups of the SAMs can have basic or acidic character and therefore allow for pH-dependent control of the surface charge. As proteins also possess a charged surface, electrostatic adsorption to charged surfaces is often strong, and the effect can be exploited for immobilization. The advantage here is that no change of the native structure of the enzyme is needed for immobilization.^20^ However, SAM-enzyme interfaces remain rather poorly understood, and the nonplanar, defect-rich structure of the underlying npAu carrier electrode introduces another degree of intricacy to the system. Accordingly, the present study aims at unveiling the effects of the electrode surface charge and porosity on enzyme activity. Working with charge interactions, the presented strategy may be transferable to various enzymes.

**Figure 1:**
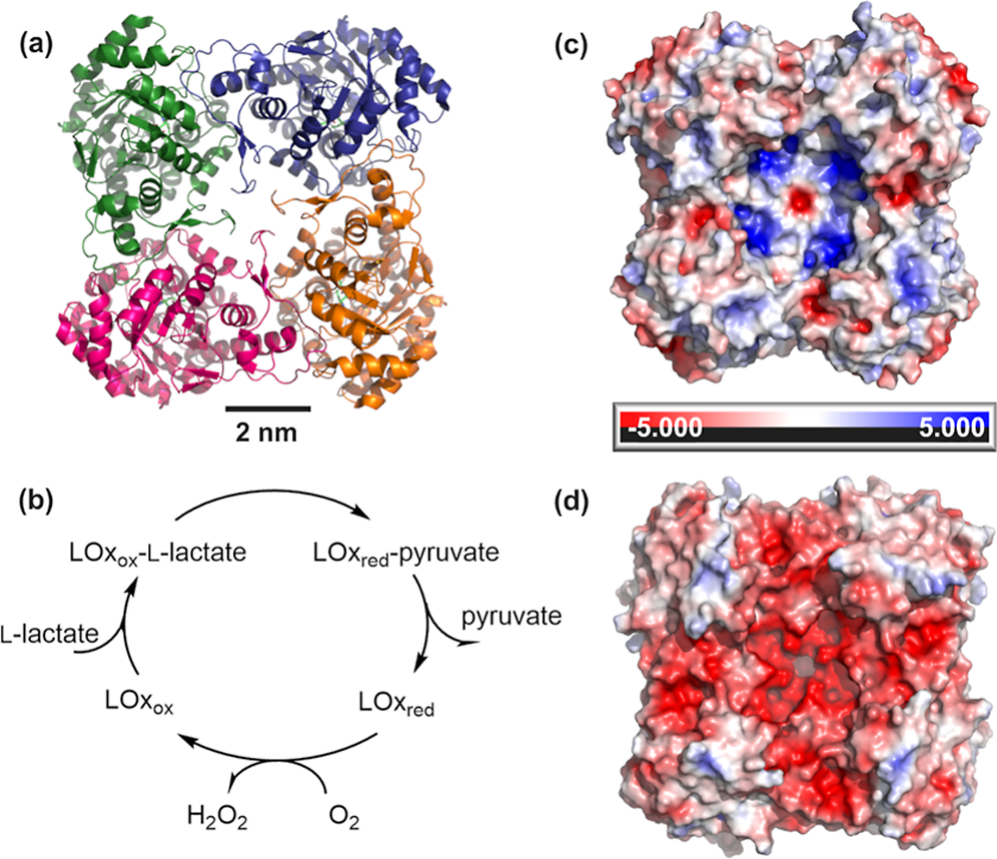
Different representations of LOx and the reaction catalyzed by the enzyme. (a) Crystal structure of LOx (PDB code. 2e77). (b) LOx reaction scheme. For details, see main text. (c) Simulation of the surface charge distribution of the front side and (d) back side of LOx. Charges are colored red (negative) to blue (positive). Protein structures are visualized in PyMOL Molecular Graphic Systems, Version 2.6 Schrödinger, LLC. Charges were calculated and color scale-represented by APBS software.^27^

Here, *Aerococcus viridans*l-lactate oxidase (for simplicity hereafter termed LOx) was selected as a model enzyme for investigation. LOx is a widely utilized enzyme in clinical diagnostics and food industry for the detection of l-lactate and thus has been subject to comprehensive characterization.^21,22^ LOx is a functional homotetramer with characteristic dimensions of approximately 50 × 100 × 100 Å,^23^ as illustrated in Figure 1a. It is a flavoenzyme that catalyzes the oxidation of l-lactate to pyruvate. O_2_ serves as the electron acceptor and H_2_O_2_ is released as the second product (Figure 1b).^24,25^ In sensing, the LOx reaction is commonly monitored by H_2_O_2_ but O_2_ is also used. At the typical working pH of 7.5, the overall negative surface charge of LOx (Figure 1c,d) is predicted to be −10.6.^26^ To investigate the surface charge-dependent immobilization, we analyzed electrodes modified with SAMs that harbor positively as well as negatively charged functional groups and applied biochemical and electrochemical methods to a detailed characterization of the resulting LOx electrodes. Based on evidence of immobilized oxidase activity, a positively charged amine and a negatively charged sulfonic acid-terminated SAM were selected for further investigation, with a particular focus on the dependence on the pore size of the carrier electrode. The sensitivity of electrodes toward l-lactate was assessed via chronoamperometry (CA) and compared to LOx-functionalized gold wires as the planar counterparts. Electrochemical oxidation of the enzymatically produced H_2_O_2_ at the (np)Au surface was used to monitor activity.

Overall, the presented study provides fundamental insight into charge-based enzyme-carrier interactions and demonstrates the advantages of npAu as an electrode material. The findings may therefore be applicable to the design of a broad range of porous bioelectrodes.

## 2 Experimental Methods

### 2.1 Materials

Gold (Au, 99.9%), silver (Ag, 99.9%), and platinum (Pt, 99.9%) wires were from ChemPur. The following molecules were used as SAMs: 10-amino-1-decanethiol [HS(CH_2_)_10_NH_2_, ADT] was synthesized (see Supporting Methods) as described in literature^28,29^ and characterized by NMR (Figures S1 and S2). 3-Mercaptopropionic acid (HSCH_2_CH_2_CO_2_H, MPA), 16-mercaptohexadecanoic acid (HS(CH_2_)_15_CO_2_H, MHDA), both >99%, cysteamine (NH_2_CH_2_CH_2_SH, CYA, ≥98%), and sodium 2-mercaptoethanesulfonate (HSCH_2_CH_2_SO_3_Na, MESA) were from Sigma-Aldrich. For chemical structures of the SAMs, see Figure S3. Perchloric acid (HClO_4_, >99%, 1 M) also was from Sigma-Aldrich. Potassium hydroxide (KOH, >85%), monopotassium phosphate (KH_2_PO_4_, >99%), and dipotassium phosphate (K_2_HPO_4_, >99%) were from Carl Roth. All abovementioned chemicals, apart from ADT and MHDA, were dissolved to the desired concentration in high-purity water (ROTIPURAN, Carl Roth). ADT and MHDA were dissolved in ethanol (Carl Roth, ≥99.5%). Potassium phosphate buffer (KPi, 50 mM) was prepared by titrating 50 mM KH_2_PO_4_ and 50 mM K_2_HPO_4_ to pH 7.5. Recombinant LOx was obtained from Roche GmbH as a lyophilized powder (Material No. 04822277103) and diluted to the targeted concentration of 0.5–0.7 mg/mL in KPi.

### 2.2 Protein Concentration

A NanoDrop spectrophotometer (DS-11 spectrophotometer, DeNovix) was used to determine protein concentrations before immobilization. Adsorption was measured at a wavelength of 280 nm, and the concentration was calculated with Lambert–Beer’s law using an extinction coefficient of ε = 51.340 M^–1^.^25^

### 2.3 Enzyme Electrode Preparation

Electrochemical preparation as well as electrochemical activity assays were performed at room temperature in a three-electrode setup using a commercial Ag/AgCl (3 M KCl with a 3 M KNO_3_ salt bridge) electrode as the reference, in relation to which all potentials hereafter are given. Measurements were controlled by an Autolab PGSTAT128N potentiostat using the NOVA 1.11 software. An Au-Ag (25 at %/75 at %) master alloy was prepared by arc melting and homogenization at 800 °C in an argon atmosphere for 12 h. It was rolled to a thickness of 120–150 μm and finally annealed in a vacuum oven at 10^–6^ mbar and 600 °C for 1 h. The alloy was cut in rectangular pieces of approximately 6 mm × 4 mm and contacted by a gold wire, which was wrapped around them. The nanoporous gold electrodes were obtained by potential-controlled dealloying, using a Pt-wire counter electrode (CE). The CA process was performed at 1100 mV in 0.1 M HClO_4_ until the current had fallen below 50 mA, yielding pore sizes of 10–20 nm.^30,31^ After electrochemical reduction of the primary oxide^32^ (for details, see a previous publication of our working group^12^), the platelets were thermally coarsened at different temperatures for 2 h in a vacuum oven to obtain the desired pore sizes of up to 400 nm. The electrochemically active surface area was determined by measuring cyclic voltammograms (CV) in the double layer regime (Figure S4). Assuming a specific double layer capacity of 40 mF cm^–2^ in HClO_4_^33^ and making use of the current dependence on the scan rate, the specific surface area as well as the mean pore size were then calculated, as explained in more detail in the Supporting Information (see section Electrochemical Pore Size Determination).

The different SAMs were applied by immersing the npAu samples in 5.0 mM aqueous (for MPA, CYA, and MESA) or ethanol (for ADT and MHDA) solutions for 72 h. Afterward, unbound molecules were removed by soaking in high-purity water (1 h). Successful modification was checked by CV in strong alkaline media, using a carbon cloth CE (Figure S5).

LOx was immobilized by immersing the differently functionalized electrodes in 0.5–0.7 mg/mL enzyme solution for 16 h at 4 °C. Afterward, the obtained bioelectrodes were washed by soaking in 1 mL of KPi for 1 h at the same temperature.

For investigating the influence of the geometric outer surface of the rigid metal carrier, crumbled samples were obtained by smashing (SAM-modified) npAu electrodes prior to LOx immobilization in the corresponding containers. Upon crumbling, the pores and SAMs remained intact, while the share of the outer surface area of the samples was increased. LOx was immobilized as described above by adding the enzyme solution to the sample. The supernatant was removed with a pipette, followed by short rinsing of the samples with KPi. Finally, 1 mL of KPi was added as the washing buffer for 1 h.

### 2.4 Electrochemical Activity Assay

All electrochemical activity assays were performed in KPi using npAu or gold wire (Au-wire) working electrodes (WEs) with LOx immobilized on different SAMs.

CVs were recorded in 10 mL of KPi at a scan rate of 5 mV/s between −850 and 200 mV upon continuous stirring at 350 rpm. A curled Pt-wire served as a CE. After a defined number of cycles, 5.0 mM l-lactate was added, resulting in a current change due to the H_2_O_2_ produced by the immobilized enzyme. Following the same procedure, 20 mM H_2_O_2_ was added for the gold wires without enzyme immobilized. The onset of the H_2_O_2_ oxidation was used to identify the potentials needed for the CA activity characterization, yielding *V*_npAu_ = 250 mV for npAu and *V*_Au-wire_ = 550 mV for the Au-wire (Figure S6).

For CA, the current was measured while the corresponding fixed potential was applied to the WE, using a gold wire as CE. The reaction volume of 5.0 mL was stirred at 350 rpm for 1 h, and afterward, as soon as the current reached a stable value, the measurement was started. Upon the stepwise addition of l-lactate in the desired concentrations, LOx releases H_2_O_2_, which is then oxidized at the gold electrodes surface.^21^ The resulting current is directly proportional to the amount of H_2_O_2_ and therefore to the amount of l-lactate added to the electrolyte. During l-lactate addition, the electrolyte was again stirred at 350 rpm for 20 s to ensure a homogeneous distribution of the substrate.

### 2.5 Oxidase Activity Assay

**Figure 2:**
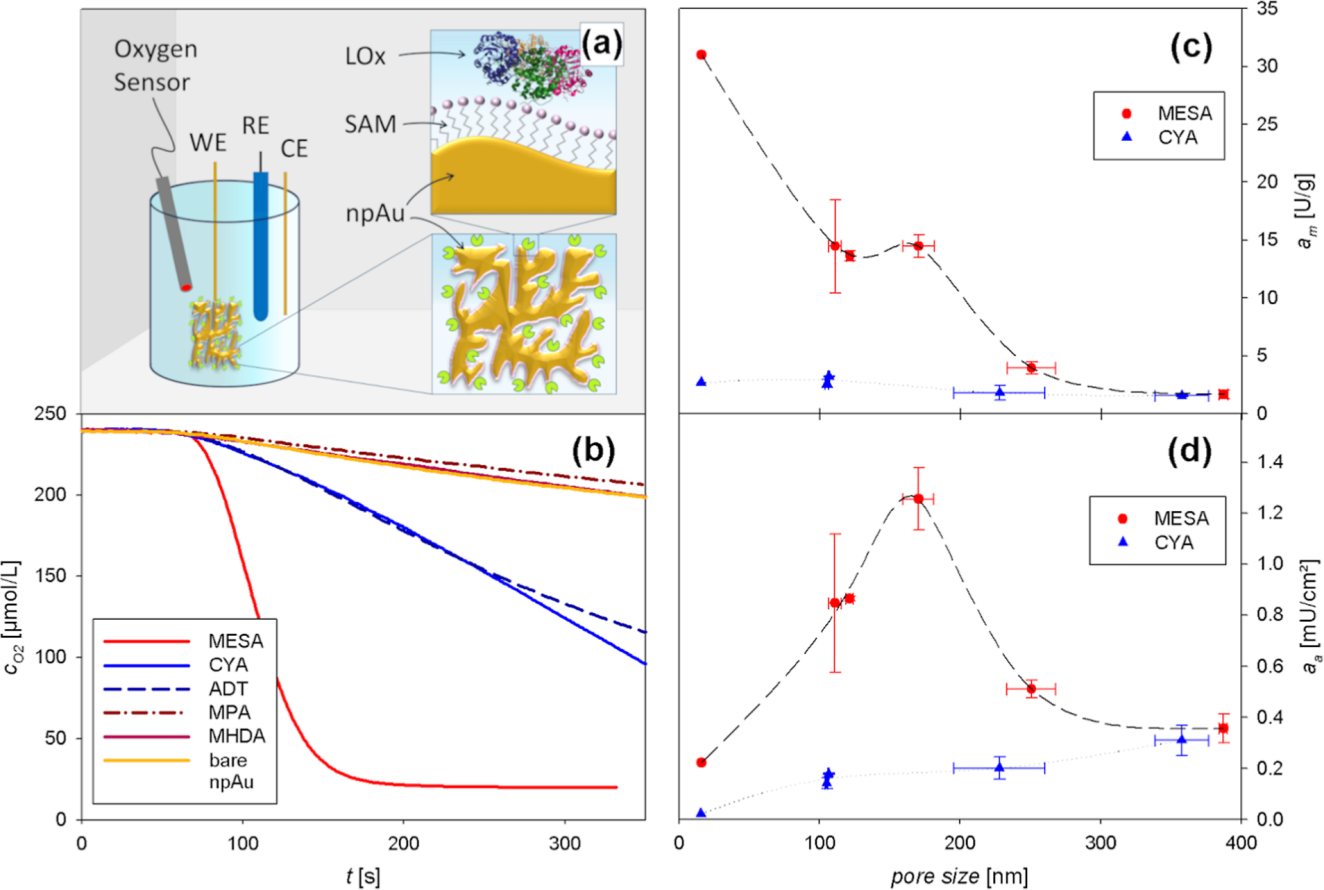
Measurement setup and enzyme electrode activity. (a) Schematic representation of the npAu-LOx electrode and setup for its characterization. RE—reference electrode, CE—counter electrode, and WE—working electrode. (b) Oxygen consumption of npAu-LOx electrodes in 50 mM KPi buffer (pH 7.5) upon addition of 5.0 mM l-lactic acid after 60 s equilibration time, using bare npAu (yellow) and several different SAMs on npAu: MESA (red), CYA (blue), ADT (dark blue, dashed), MPA (purple, dash-dotted), and MHDA (dark purple). (c) Pore size-dependent activity of the npAu-LOx electrodes with CYA (blue triangles) and MESA (red circles), normalized to the total surface area (*a*_s_ in mU/cm^2^) and (d) to the mass (*a*_m_ in U/g) of the npAu samples. Each point represents *N* measured samples (*N* = 2 or 3), except for 15 nm pore size (single measurements). Vertical error bars correspond to the standard deviation of the activity; horizontal error bars indicate the corresponding pore size range. Dashed lines represent spline fits as a visual guide.

The oxidase activity of soluble and immobilized enzymes was determined from the oxygen consumption rate. Measurements were performed according to literature,^34^ using a fiber-optic oxygen meter (FireStingO2, PyroScience). The sensor performs an optical (luminescence emission) measurement to determine the concentration of the O_2_ in the bulk solution. The sensor does not consume the O_2_. Calibration (Figure S7) was performed in air saturated (100% O_2_) and degassed (0% O_2_) KPi buffer prior to activity determination. Assays were carried out in a reaction mixture (2.0 mL) containing 5.0 mM lactate in 50 mM air-saturated KPi (pH 7.5). The mixture was stirred at 24 °C and 350 rpm, and the reaction was started either by addition of 2.0 μg of free enzyme or by inserting the enzyme electrode into the mixture. In the latter case, the sample was placed in close environment of the sensor tip (see Figure 2a). One unit (U) is defined as the amount of LOx that consumes 1 μmol of O_2_ per minute under the conditions used. The free enzyme showed a specific activity of 103 U/mg (±8%, number of measurements *N* = 5) in solution. For the activity of the enzyme electrodes, the enzymatic units were normalized to either the surface area (*a*_a_ in mU/cm^2^) or the mass (*a*_m_ in U/g) of the nanoporous gold sample. Oxidase activity assay was also used to determine the immobilization yield (*Y* in %; eq 1) and the catalytic effectiveness (*E* in %; eq 2).
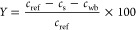
1
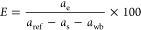
2

The symbols *c* and *a* are the volumetric activity (in U/mL) and absolute activity (in U), respectively. Subscripts ref and s correspond to the supernatant before and after immobilization, respectively, wb to the washing buffer, and subscript e to the enzyme electrodes.

## 3 Results and Discussion

This section commences with the verification of the successful modification of npAu with different SAMs, followed by an investigation of the impact of the carrier’s surface charge and pore size on LOx activity. Additionally, models for the immobilization mechanisms of LOx on the different SAM surfaces will be presented. Finally, the electrochemically examined l-lactate sensitivity of the npAu-LOx electrodes is compared with that of planar samples.

### 3.1 Self-Assembled Monolayer Modification of Nanoporous Gold

The surface coverage of the different SAMs on npAu is an important property for further enzyme immobilization. The SAM precursor molecules used in this study were selected according to the isoelectric point (IEP), which determines the surface charge of the resulting layer at a given pH value. Positively charged surfaces can be created by amine terminated molecules (ADT, CYA), whereas carboxylic acid (MHDA, MPA) and sulfonic acid (MESA) end groups result in a negative surface charge at pH 7.5. In Figure S3, IEPs of the molecules in solution are given; however, they might differ from their counterpart IEPs of the molecules bound to a surface. The high density of surface groups in a closely packed SAM layer seems to suppress the natural acid–base chemistry. Strong acids, such as MESA, still get fully deprotonated at neutral pH and thus exhibit a strong negative surface charge.^35^ Especially for weak acids and bases, the degree of dissociation and thus the surface charge can be reduced (e.g., carboxylic acids^36^ or amines^35^). On npAu, the surface coverage with SAMs is approximately 0.6 monolayer, as will be discussed in the following. Thus, the density of surface groups and therefore also the shift of the IEP might be reduced. The formation of the SAMs was verified by electrochemical desorption studies under alkaline conditions. Cyclic voltammetry (CV) was performed in 1 M KOH, showing pronounced oxidation peaks in the first cycle, which corresponds to detachment of the SAM for all molecules used in this study (Figure S5). The position of the oxidation peak maximum was found between 230 and 270 mV for the shorter-chain SAMs (MPA, CYA, and MESA, Figure S5b–d). For the SAMs with longer alkyl chains ADT and MHDA, the oxidation peak shifted to more positive potentials of 430 and 460 mV, respectively (Figure S5e,f). This shift in desorption potential with increasing chain length can be attributed to the increased stability of those molecules resulting from stronger hydrophobic interactions of the alkyl chains.^37^ The charge ratio of the SAM desorption peak with respect to the oxidation peak of the corresponding bare npAu sample was calculated. The ratio is proportional to the share of surface initially coated by the SAM and therefore represents the coverage. A value of approximately 0.6 monolayers was obtained, indicating a similar coverage for all of the different molecules used. This finding is consistent with the SAM density of greater than 60% on gold nanoparticles observed by Love et al.,^38^ who attribute the high coverage per gold atom to the large number of surface defects such as kinks and edges on the nanoparticles compared to planar substrates. Those low coordinated gold atoms are also abundant in npAu,^39^ permitting a direct comparison. Additionally, the obtained surface coverage values are in good agreement with previous work by our group: SAM formation and detachment from npAu were monitored for cysteine and MESA by in situ resistometry, and the results were correlated with electrochemical desorption measurements.^12,17^ Summarizing, the formation of the different selected SAMs was found to be successful. All monolayers exhibited similar surface coverage independent of their chain length, thereby eliminating any potential confounding factors in subsequent activity measurements.

### 3.2 Dependence of LOx Activity on Surface Characteristics

Effects of the carrier’s surface properties such as charge and pore size on the immobilization behavior of LOx were examined using the oxidase activity assay. For this purpose, the enzyme electrode’s oxygen consumption was measured upon lactate addition (setup shown in Figure 2a) and used to calculate the activity (for details, see the Experimental Methods section). The influence of both the surface charge and the chain length of the differently terminated SAMs (Figure S3) was studied at a constant pore size. Based on the results, MESA and CYA modifications were chosen to additionally investigate geometrical effects resulting from the variation of the npAu carrier pore size in a range of 15–400 nm. The results are discussed in the following sections.

#### 3.2.1 Surface Charge Dependence

Figure 2b shows the as-measured oxygen concentration over time for the differently modified npAu-LOx electrodes. The absolute values of the curve slopes represent the oxygen consumption rate proportional to the electrodes' activity. Electrodes with SAMs that possess the same functional group (amine terminated: ADT and CYA, carboxylic acid terminated: MHDA and MPA) showed similar rates, indicating that the functional groups rather than the chain length of the SAMs are relevant to the enzyme activity. Note that the observed enzyme activity is determined not only by the enzyme bound to the electrode but also by the portion of enzyme in solution retained in the porous structure. It was thus concluded that the differences in enzyme electrode activity can be attributed to the surface charge of the SAMs as a central factor. The carboxylic acid-modified and bare npAu samples exhibited flat slopes with resulting activities below 1 U/g. Modification with amine-terminated SAMs increased the electrode activity (around 3 U/g). However, the highest activity of approximately 14 U/g was observed for samples modified with the sulfonic acid-terminated MESA (Figure 2c), which has the most negative surface charge of the SAMs tested.^35^ This result was unexpected as it appears to counter the idea of repulsion of like charges: the modified metal surface exhibits the same negative net charge as LOx (Figure 1). Interestingly, the effect was seen only for the sulfonic acid SAM (MESA) while it was absent when using the less negatively charged carboxylic acid SAMs (MPA, MHDA). To further explore adsorption on like-charged surfaces, we performed LOx immobilization on polymethacrylate carrier beads with amine and sulfonic acid surface functionalization (see Supporting Methods, section Enzyme Activity on Polymer Carriers). The results from this additional study were coherent with expectations for LOx immobilization by electrostatic interactions.^25^ The amine-modified beads showed a more than 7-fold higher activity (27 U/g) than the sulfonic acid-modified ones. Considering the material density of gold (19.3 g/cm^3^)^40^ and polymethacrylate (1.2 g/cm^3^),^41^ the sulfonic acid-modified npAu-LOx electrodes showed 9 times higher activities per mass than LOx on the amine-modified beads. As no increased LOx activity was found on sulfonic acid-functionalized polymer beads, this effect was considered specific for the metal carrier, and a possible dependence on its pore size was examined. For a comparison with the MESA-modified npAu-LOx electrodes of different pore sizes, bare and amine-modified surfaces were also considered in the following studies. Here, the CYA SAM was chosen as the molecule has the same carbon chain length as MESA and is therefore ideally comparable.

#### 3.2.2 Pore Size Dependence

The activity of the npAu-LOx electrodes discussed in the following section is always related to the electrochemically determined mean pore size of the corresponding sample. Of course, not all pores and ligaments in the self-similar structure exhibit the same size, but rather follow some pore size distribution. Therefore, scanning electron microscopy (SEM) was performed to additionally analyze the porous structure of the npAu samples (Figure S8). A number of studies in the relevant literature have already been published. Bapari et al.^42^ investigated the pore sizes for as-dealloyed as well as thermally annealed samples and found a good agreement between values obtained by electrochemistry and by SEM. The standard deviation of the pore size in individual samples was found to scale with the mean pore size by a factor of approximately 0.3 and is not affected by coarsening. The SEM images presented in their work compare well with those of our npAu samples, and thus, the quite narrow normalized standard deviation is also assumed in the present study.

Figure 2c,d shows the activities of various npAu-LOx electrodes modified with MESA and CYA, exhibiting pore sizes in the range of 15–400 nm. The activities of the single samples were calculated from the oxygen measurements (exemplarily shown in Figure 2b) and combined to sets with a number of *N* = 2–3 samples with similar pore sizes. Figure 2c shows activity values normalized to the mass, while Figure 2d shows normalization to the electrochemically active surface area of the npAu samples. The difference between the values of the activity per surface area and per mass can be attributed to the high specific surface area of the samples. While the mass decreased by approximately 62%, the npAu electrodes surface area increased by a factor of 30–6000 after dealloying and thermal treatment, depending on the final pore size, explaining the smaller values for the activity per area. In both normalizations, to the mass and the surface area, samples modified with MESA demonstrated enhanced activity compared to those with CYA across the range of pore sizes examined. The surface dependent activity of the CYA electrodes increased from 0.02 mU/cm^2^ at a pore diameter of 15 nm to a highest value of 0.31 mU/cm^2^ at 400 nm, while the mass-dependent activity had no distinct pore size dependence. The deviations between the individual samples (apparent by the error bars) were below 30%. In contrast, the activity of the MESA samples demonstrated a pronounced pore size dependence, with variations up to 90%. Figure 2c shows an increasing activity normalized to the sample mass with decreasing pore size using MESA. Moreover, a peak is observed at pore sizes of (170 ± 11) nm. This peak is visually even more pronounced when the activity is normalized to the surface area of the samples (Figure 2d), which is inversely proportional to the pore size (see Supporting Information, section Electrochemical Pore Size Determination). This peak in the activity appears to be caused by the interplay of different pore size-dependent effects. At decreasing pore sizes to ∼170 nm, the activity per unit mass and per surface area increase. This phenomenon may be attributed to two distinct effects. First, the surface area and, consequently, the quantity of immobilized enzyme increases, resulting in an enhanced activity (per mass) of the enzyme electrode.^9^ Second, enzyme diffusion out of the porous network during the washing step is more hindered the tighter this network becomes. The decline in activity observed at pore sizes below approximately 160 nm is attributed primarily to limitations in l-lactate and/or O_2_ diffusion limitations,^43,44^ as explained in the following. Bolivar et al.^45,46^ demonstrated that for different enzymes immobilized on polymer beads, the concentration of O_2_ inside the porous structure drops immediately after the start of the enzymatic reaction. This results in the O_2_ supply becoming the rate-limiting step of the overall enzymatic conversion. The authors proposed that the O_2_ is used up already slightly below the carrier’s surface, preventing the transport to the inside of the porous network and resulting in a limited number of enzymes participating in the reaction. This concept appears to be applicable to npAu carriers as well, and a comparable phenomenon may occur with lactate. As the pores become smaller, diffusion processes are also more constrained,^15,47^ which may lead to the observed decline in activity with decreasing pore size as shown in Figure 2d.

Besides O_2_ diffusion, diffusion of the enzyme into the porous structure can also be hindered during the immobilization process. Enzymes already adsorbed in the outermost layers or close to a pore entrance can form barriers for following enzymes and slow down adsorption. However, this effect is stronger, the closer the pore size comes to the diameter of the enzyme.^9,48^ At 15 nm pore size, just one single event of LOx adsorption might lead to pore clogging, and thus, immobilization inside the porous structure is impeded by enzyme diffusion.^17^ For all other samples with pore sizes larger than 100 nm, the enzyme should be able to enter the porous network.^9^ The timescale of the immobilization process (16 h) is assumed to be sufficient for LOx to reach the inner layers of the electrodes.

**Figure 3:**
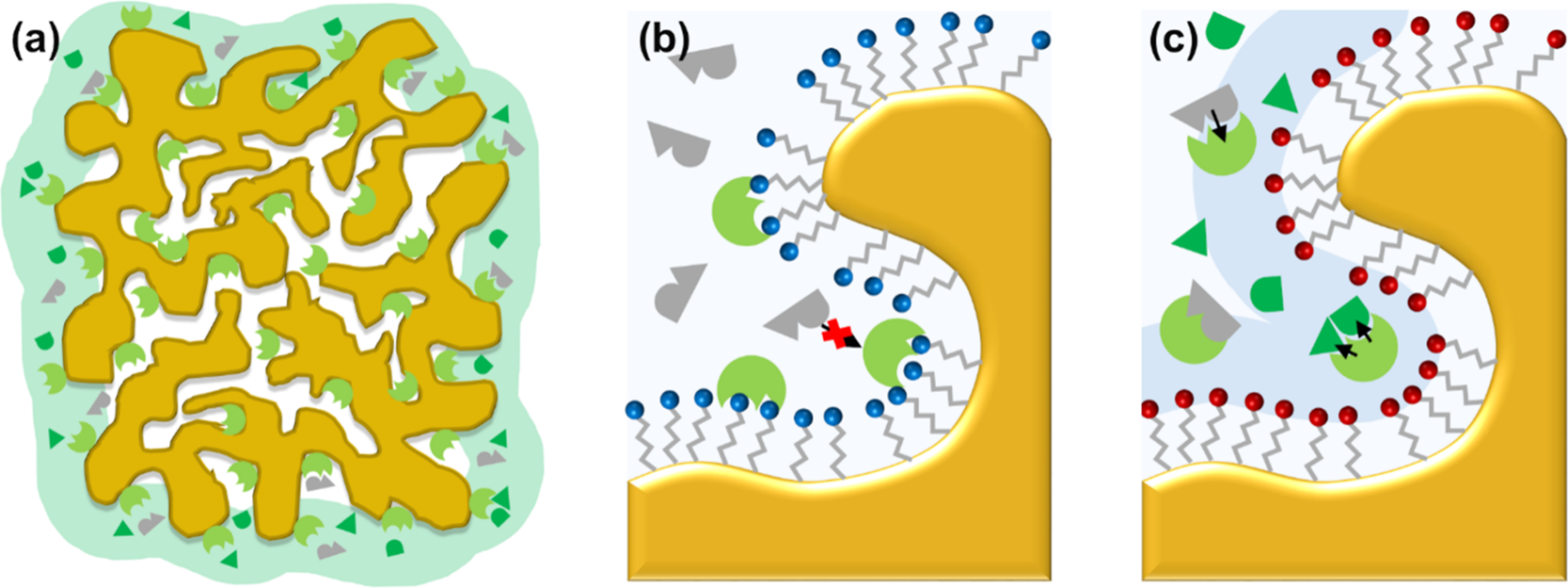
Illustration of the activity of different npAu-LOx electrodes. (a) Only enzymes (light green) immobilized close to the surface take part in the catalytic reaction, while the O_2_ and l-lactate (gray) do not reach enzymes inside the bulk porous structure due to diffusion limitations. (b) The electrostatic binding of LOx on a CYA (blue) modified surface seems unfavorable for the activity of the enzyme. (c) MESA (red) modified npAu sample where LOx is captured in a layer of counterions (light blue) and converts substrate into product (dark green).

Table 1 shows one set of enzyme electrodes with pore sizes between 100 and 150 nm, which is in the range that results in high activity per mass (*a*_m_). The samples were prepared concurrently under identical conditions except for the SAM modifications, allowing for a comprehensive and detailed comparison. LOx immobilization on bare npAu yielded the lowest activity. As described above, surface modifications of the carrier electrodes increased the activity *a*_m_ more than 4 times for CYA and 18 times for MESA. The immobilization yield was similar for both surface modifications with ∼50% and highest for the bare samples with approximately 83%. The catalytic effectiveness *E* followed the same trend as that of *a*_m_ for the different electrode types. The low value of *E* below 6% for all samples is most likely also a consequence of the restricted oxygen supply within the porous network. The proportion of enzymes with sufficient O_2_ and substrate supply for catalyzing their reaction is probably limited (as schematically shown in Figure 3a). However, the effectiveness factor *E* considers all of the immobilized enzymes. With an activity of 14 U/g, extrapolation of the data obtained by Bolivar et al.^45^ would result in an *E* value of approximately 10%. However, the earlier study^45^ used polymer beads with a particle size of 100–300 μm and pore sizes of 10–20 nm, where the ratio of outer to inner surface area is higher than for the bulk npAu used in this work. As discussed above, only enzymes close to the outermost surface of the carrier may participate in the catalytic reaction. Therefore, a smaller share of outer surface for the npAu carriers may lead to a smaller number of active enzymes and therefore lowered *E* values.

**Table 1:** Activity per Mass (*a*_m_), Immobilization Yield (*Y*), and Catalytic Effectiveness (*E*) of npAu-LOx Electrodes with Different Surface Modifications^a^

surface modification, pore size [nm]	*a*_m_ [U/g]	*Y* [%]	*E* [%]
none, (130 ± 24) nm	0.7 ± 0.1	83 ± 4	0.23 ± 0.04
CYA, (106 ± 1 nm)	2.9 ± 0.4	49 ± 5	1.23 ± 0.40
MESA, (118 ± 6 nm)	13.0 ± 1.0	50 ± 7	5.27 ± 0.79

a All samples (*N* = 3 for each type) are from one experimental set, using a stock solution of 0.5 mg/mL LOx for immobilization.

The high immobilization yield for the bare gold electrodes is a result of the low activity of supernatant and washing buffer compared to the reference. Presumably, LOx is getting denatured at the bare gold surface.^16^ This causes a small activity of the enzyme electrodes, and no active enzyme gets eluted into the washing buffer. Additionally, some free enzyme in the supernatant might denature due to contact with the metal surface during the immobilization process, causing a low activity of this solution as well.

The presented measurement series shows that the catalytic effectiveness of the CYA samples is below that of the MESA samples. The more than 4 times lower *E* values suggest additional activity decreasing effects of the amine-modified surface, besides the O_2_ diffusion limitations. This assumption is consistent with previous work of Luley-Goedl et al.,^25^ in which LOx was immobilized on nonconductive porous agarose carriers with amine modification. Due to an effectiveness factor of 0.23, they assumed that conformational changes upon electrostatic adsorption might lead to sterically hindered or even inactive enzyme.^49,50^ It was demonstrated^25^ that after desorption, LOx had the same activity as before immobilization, indicating that adsorption does not result in irreversible structural modification of the enzyme or denaturation. We suggest a similar phenomenon for LOx on amine-modified npAu (Figure 3b). The 18 times higher *E* value of enzyme immobilized on the polymer carriers can again be explained by the increased share of the outer surface area, which is even higher for a particle size of 40 μm and pore sizes between 20 and 40 nm as used in the earlier work.^25^

#### 3.2.3 Proposed Immobilization Mechanisms

As shown in Table 1, MESA and CYA samples showed an immobilization yield of approximately 50%. Still, a four times higher activity and catalytic effectiveness were seen when using MESA. The improved characteristics of the npAu-LOx electrodes suggest a more favorable position of the LOx with respect to the MESA-modified surface. As attractive electrostatic interactions between the negatively charged LOx and a like-charged solid surface can be excluded, another form of immobilization of the enzyme is assumed to take place on npAu-MESA. A tentative explanation is that the LOx is captured in an electrochemical double layer of ions that is formed as result of the charged gold surface being in contact with an electrolyte (potassium phosphate buffer). The general effect is well-known: counterions of the liquid phase become enriched in the liquid layer adjacent to the surface to neutralize the surface charge. Evidently, the higher the surface charge, the greater is the number of ions required to balance it.^51^ We envision a scenario in which the LOx (a protein polyelectrolyte of negative net charge) is attracted to the positively charged ion layer formed in close proximity to the MESA-coated surface of npAu (Figure 3c). The LOx may be bound loosely within the charged layer adjacent to the solid surface. The absence of a direct interaction of the enzyme with the solid surface may contribute to the retention of activity. We also note the possibility of overcharging of the electrochemical layer. This results when counterions of the charged surface groups accumulate in excess within the surface layer of solvent, thus leading to charge inversion.^52,53^ Taking the experimental value of ∼0.6 for the monolayer surface coverage of npAu with MESA, we can apply literature procedure^54^ to obtain an estimate of approximately 0.5 C/m^2^ for the surface charge density of the MESA electrode used. This value is within the range of surface charge densities revealed in simulation studies by Agrawal et al.^55^ on the surface phenomenon of overcharging. Overcharging could arguably benefit the capture of LOx in the electrochemical layer of the npAu-MESA.

In order to ascertain the interaction between the enzyme and excess cationic charge in the electrochemical double layer, npAu-MESA electrodes were immersed in LOx solutions with KPi concentrations *c*_KPi_ of 10, 50, and 100 mM. The washing buffers and the buffer utilized in the oxygen activity assay were also at the respective concentrations. Table 2 shows the resulting activity per mass (*a*_m_) and per surface area (*a*_s_). For *c*_KPi_ = 50 mM, the activity of the samples is in the same range as for the other samples at this pore size (approximately 385 nm, see Figure 2c,d). Increasing *c*_KPi_ to 100 mM resulted in a 4-fold increase in the activity of the npAu-LOx electrodes, while for the 10 mM buffer, only one-third to one-half of the activity was measured. The hypothesis of enzyme capture in the double layer is substantiated by these activity changes with buffer concentration since increasing the buffer concentration also increases the ionic density of the double layer and thus should result in stronger interactions with the LOx.

**Table 2:** Activity per Mass (*a*_m_) and per Surface Area (*a*_s_) of npAu-MESA-LOx Electrodes in Dependence of the KPi Buffer Concentration (*c*_KPi_)^a^

*c*_KPi_ [mM]	*a*_m_ [U/g]	*a*_s_ [mU/cm^2^]
10	0.57 ± 0.01	0.10 ± 0.00
50	1.33 ± 0.06	0.29 ± 0.01
100	4.95 ± 0.62	1.20 ± 0.14

a All samples (*N* = 3 for each type) are from one experimental set, using stock solutions of 0.5 mg/mL LOx in KPi with the different concentrations for immobilization. All samples used exhibited mean pore sizes of (385 ± 18) nm.

The relatively larger error in the activity measured for the LOx immobilized on npAu-MESA (Figure 2c,d) aligns with the hypothesis of loose enzyme capture in the surface layer of the solvent. The weak nonspecific interactions involved in the capture of the enzyme in this layer could also result in a higher degree of variability in the results. This is due to experimental variations over different sets such as those associated with immobilization and washing times.

We also considered the possibility that the LOx adsorbs to npAu-MESA in a preferred molecular orientation so that a positively charged surface region of the protein interacts with the surface.^55−57^ However, high activity of the immobilized LOx was not observed with the carboxylate-terminated SAMs (Figure 2b), which also creates a negatively charged surface. It is therefore assumed that the LOx is electrostatically repelled by those SAMs, which is inconsistent with the idea of enzyme adsorption. Due to their higher IEP (see Supporting Information, Figure S3), the carboxylate groups are not as strongly charged in an electrolyte solution of pH 7.5 as the sulfonate groups of MESA, and thus, less positive charge is required to balance their surface charge. The ion layer formed at the surface is probably not dense enough to capture the enzyme. Also, overcharging may not occur at weakly charged surfaces.

### 3.3 l-Lactate Sensitivity

**Figure 4:**
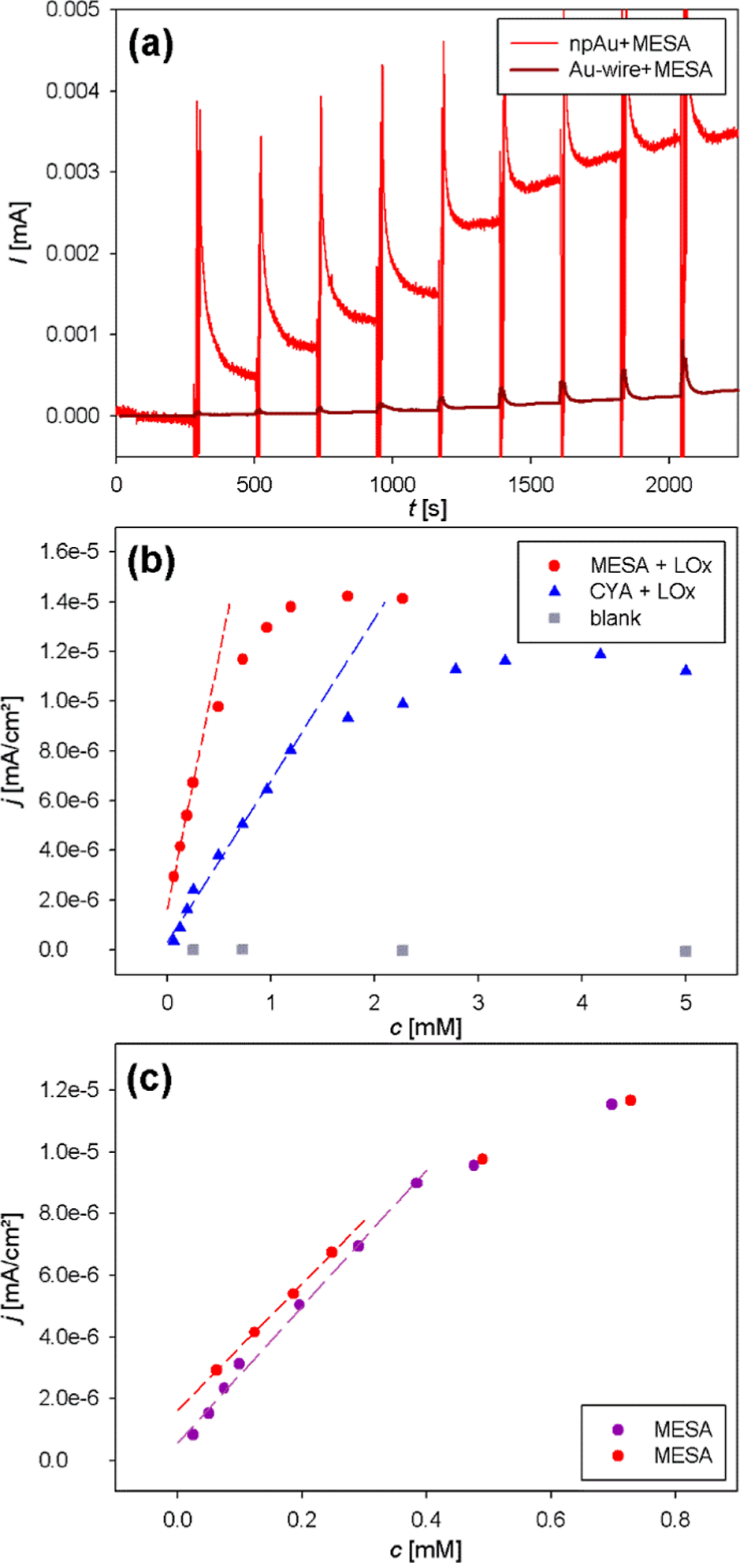
Electrochemical l-lactate sensing. (a) CA of a npAu (red) and Au-wire (dark red) with MESA and LOx. Current steps correspond to l-lactate addition with concentrations equal for both samples. (b) Mean current density *j* over l-lactate concentration *c* as determined from CA. npAu samples were modified with MESA (red circles) or CYA (blue triangles) and LOx. A blank measurement (gray squares) was performed using npAu-MESA without enzyme. (c) Two different npAu-MESA-LOx electrodes. The sample plotted in red is the same as in subfigure (b); for the sample plotted in purple, l-lactate was added in smaller concentrations. Dashed lines represent fits of the linear parts.

We next examined the enzyme electrodes regarding their use for l-lactate sensing based on a CA activity assay. The H_2_O_2_ released by the LOx reaction is measured. To additionally investigate the influence of the carrier geometry, nanoporous electrodes were compared to planar ones modified in a consistent manner. The electrochemical measurement setup using three-electrode geometry is shown in Figure 2a. As visible in Figure 4, each l-lactate addition step was characterized by an immediate current change. The peaks in the beginning of each current step (Figures 4a, S7, and S8) were caused by the electromagnetic field of the stirrer switched on during the addition of the l-lactate. The calculated mean current densities are shown in Figure 4b,c; for original CA data, see Supporting Information (LOx: Figure S9, blank: Figure S10).

The current increase as well as the absolute value of the current was 10 times higher for the npAu sample than for the Au-wire (Figure 4a). Both effects presumably arise due to the enlarged surface area of npAu compared with that of the planar gold wires. The larger surface area allows for the immobilization of a greater number of enzymes, thereby producing a greater quantity of H_2_O_2_ at steady state. As an additional effect, the SAM density of less than one monolayer on npAu^12^ provides a greater portion of bare gold surface for the H_2_O_2_ conversion process to take place, which can further increase the current signal. The nanoporous electrode required a 300 mV lower potential for H_2_O_2_ oxidation (see Experimental Methods, section Electrochemical Activity Assay) and therefore showed a reduced energy requirement. This is due to the intrinsic electrocatalytic activity of the material caused by its defect and curvature rich structure.^56^ In comparison to planar Au(111) surfaces, npAu also provides enhanced SAM stability due to the high number of low-coordinated surface atoms and residual silver atoms.^57^ This was also verified here by desorption studies via CV (Figures S11 and S12), showing the stability of the SAMs during CA for npAu but not for the planar gold wires.

Figure 4b,c shows the mean current density *j* per l-lactate addition step for different npAu samples. The initial increase of *j* with l-lactate concentration exhibits a linear behavior up to 0.4 mM for MESA and 1.2 mM for CYA-modified npAu-LOx electrodes (Figure 4b). The obtained sensitivity yielded 1.6 μA mM^–1^ for the CYA-modified sample and a 2.5-fold higher value of 3.9 μA mM^–1^ for the MESA-modified sample. For the planar samples (Au-wire), the sensitivity using a MESA modification was higher than that for CYA as well (approximately 1.7-fold, Figure S13). This shows that the effect of enzyme capture close to the MESA surface is less pronounced but also visible for nonporous metal carriers.

The current density of the MESA sample from Figure 4b is compared to that of another npAu-MESA-LOx electrode in Figure 4d. For the second sample, smaller l-lactate concentrations (starting at 25 μM) were added. Still, the current density of the two samples changed with a similar slope, yielding sensitivity values of 3.9 μA mM^–1^ and 3.3 μA mM^–1^, thus highlighting good reproducibility.

The sensitivity values (in μA mM^–1^) obtained for the npAu-MESA-LOx electrodes are well within the range of gold-based l-lactate sensors documented in literature,^22,58,59^ showing the significance of the bulk npAu carrier and the chosen modification by SAMs for enzyme electrode design. Nevertheless, a detailed comparison of the sensing properties of the npAu-MESA-LOx electrodes with existing porous gold-based l-lactate sensors using LOx is challenging. This is due to the diverse range of geometries that are characterized as nanoporous gold electrodes. Many of these electrodes have only a few pore/ligament layers,^58,60^ whereas the samples in this study represent a bulk porous network. The advantage of these bulk samples is, however, that they can be prepared directly as no additional materials are necessary for the design of the electrodes.

### 3.4 Binding Study of LOx

**Figure 5:**
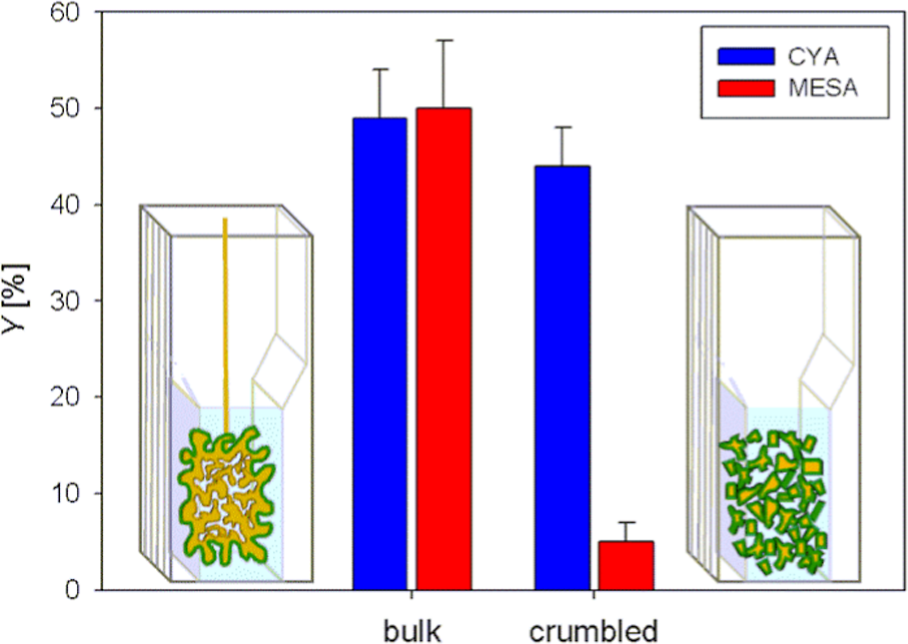
LOx immobilization yield (*Y* in %) on CYA or MESA-modified bulk (left) and crumbled (right) npAu samples. The directly accessible outer surface is shown in dark green in the schematic representations.

The proposed mechanism of ion layer capture of LOx for immobilization on npAu-MESA implies an important contribution from the nanoporous network structure of npAu to the retention of enzyme activity by the solid material. Diffusion into the npAu sample is slow (time domain of several hours), as shown in earlier work.^15^ Thus, washing with buffer is expected to release the immobilized LOx only from areas of the carrier surface that are accessible for solvent layer exchange within the time of the experiment (1 h). We hypothesized that LOx captured in the ion layer of the npAu-MESA would be much more easily removed from smaller particles of overall lower internal porosity. By contrast, the removal of enzyme immobilized by surface adsorption (npAu-CYA) would be less sensitive to the bulk structure of the material. Thus, mechanical crumbling of the bulk npAu sample was used to demolish the self-standing nanopore structure of the material to generate the desired smaller npAu pieces as shown in the schematic in Figure 5.

The bar chart shows the immobilization yield calculated from the supernatant and washing buffer activity (eq 1). For LOx on CYA-gold, the yield was very similar to 44 and 49% for crumbled and bulk samples, respectively. The result is consistent with the notion that the enzyme exhibits robust electrostatic adsorption on the positively charged surface. In contrast, the supernatant and the washing buffer from the crumbled samples showed increased activities when an MESA modification was employed. This result indicates a reduced capture and accelerated release of the LOx. The two effects in combination lead to an immobilization yield that is approximately ten times lower than that for the bulk samples. Thus, the idea of a loose capture of the LOx on the MESA surface within the nanoporous network of the bulk npAu sample is supported.

To further test the binding strength, LOx elution from bulk npAu-LOx electrodes immersed in washing buffer was studied over a period of 6 days. The activity of each sample was subsequently monitored via an oxidase activity assay. Results showed a significantly faster decrease for the MESA samples (Figure S14). The decrease was especially pronounced for the first washing step (∼half a day) with around 70% for the MESA samples, while the CYA samples still exhibited around 50% of the initial value. The activity found in the washing buffers of both electrode types was in accordance with the activity lost from the solids. The electrode stability is determined by the strength of capture and binding of the LOx. However, despite the faster release of enzyme activity from npAu-MESA, the absolute activity was still 2.5-fold higher for the MESA samples than for the CYA samples over the entire time span. This again indicates a more favorable accommodation of the LOx on npAu-MESA, despite the less stable adsorption. Therefore, a future step will be to improve the stability of the enzyme on the MESA-modified npAu surface and avoid elution. One approach could be to form a protective layer around the electrode after immobilization, e.g., using glutaraldehyde.^61−63^ It was, however, beyond the scope of this study.

In summary, it was found that a strongly like-charged surface can have positive effects on the activity of the npAu-enzyme electrodes. A loose capture of the LOx within the charged ion layer in the proximity of the MESA modified surface is assumed. In contrast to electrostatic adsorption on positively charged CYA surfaces, the absence of direct interaction between the LOx and a solid surface may help in retaining the enzyme’s activity.

## 4 Conclusions

The activity of the model enzyme LOx was found to be enhanced on nanoporous gold (npAu) modified with a sulfonate-terminated self-assembled monolayer (MESA). In contrast to the conventional electrostatic attraction of opposite charges, the npAu-MESA surface exhibits the same charge as that of the enzyme. We postulated that the enzyme is captured in a counterion layer at the electrode–electrolyte interface, which is known to form due to the strong negative surface charge of the electrode. The absence of direct interaction between the enzyme and the solid surface may contribute to the retention of activity. A pronounced pore size dependence of the activity of the enzyme electrode is found, which arises from the interplay between the available surface area and diffusional limitations of the nanoporous network of npAu. In contrast, the electrostatic adsorption of LOx to a positively charged surface modification (CYA) has been observed to result in a 4-fold reduction in activity, which is likely attributable to the direct binding interaction. The proposed immobilization mechanisms were supported by binding studies, with samples exhibiting varying degrees of internal porosity.

In summary, this study offers fundamental insight into charge-based interactions between enzymes and metal carriers, emphasizing the importance of selecting an appropriate pore size to achieve optimized activity. Consequently, our findings are applicable to a multitude of enzymes, thereby facilitating the design of a diverse array of nanoporous metal-based bioelectrodes.

## Supporting Information Available

Additional synthesis details and characterization, experimental methods, and supporting figures (PDF)

## Abbreviations

ADT: 10-amino-1-decanethiol [HS(CH_2_)_10_NH_2_]
Au-wire: gold wire
CA: chronoamperometry
CE: counter electrode
CV: cyclic voltammetry
CYA: cysteamine
IEP: isoelectric point
KPi: potassium phosphate buffer
LOx: *Aerococcus viridans*l-lactate oxidase
MESA: 2-mercaptoethanesulfonic acid
MHDA: 16-mercaptohexadecanoic acid
MPA: 3-mercaptopropionic acid
npAu: nanoporous gold
REF: reference electrode
SAM: self-assembled monolayer
WE: working electrode.
