# Supplementary material for: Interplay of Surface Charge and Pore Characteristics in the Immobilization of Lactate Oxidase on Bulk Nanoporous Gold Electrodes
Source: Langmuir. 2025 Feb 20;41(8):5136–46. doi: 10.1021/acs.langmuir.4c04367 (PMC11887427; doi:10.1021/acs.langmuir.4c04367)
Supplement: Supplementary file 1 — la4c04367_si_001.pdf [file la4c04367_si_001.pdf]

SUPPORTING INFORMATION

Interplay of surface charge and pore characteristics in the immobilization of lactate oxidase on bulk nanoporous gold electrodes

*Lara Marie Novak[1], Elisabeth Hengge[2], Eva-Maria Steyskal[1], Roland Würschum[1] and Bernd Nidetzky[2]**

[1] Institute of Material Physics, Graz University of Technology, NAWI Graz, Petersgasse 16, 8010 Graz, Austria.

[2] Institute of Biotechnology and Biochemical Engineering, Graz University of Technology, NAWI Graz, Petersgasse 16, 8010 Graz, Austria.

*Correspondence: bernd.nidetzky@tugraz.at

Table of Contents

Supporting Methods S3

Synthesis of ADT S3

NMR Characterization S4

Electrochemical pore size determination S4

Enzyme activity on polymer carriers S5

Supporting Figures S6

Figure S1. ^{1}H NMR of ADT S6

Figure S2. ^{13}C NMR of ADT S7

Figure S3. Chemical structure of the SAMs S8

Figure S4. Electrochemical pore size determination S8

Figure S5. CVs showing SAM desorption S9

Figure S6. CVs to determine the potentials used for CA S10

Figure S7. Oxygen sensor calibration S10

Figure S8. SEM image of npAu S11

Figure S9. As-recorded CA data of gold-enzyme electrodes S11

Figure S10. Blank CA measurements S12

Figure S11. Stability of SAMs on npAu S12

Figure S12. Stability of SAMs on Au-wires S13

Figure S13. L-lactate sensitivity of Au-wire-LOx electrodes S13

Figure S14. Elution of LOx from npAu electrodes S14

References S15

Supporting Methods

Synthesis of 10-amino-1-decanethiol (ADT)

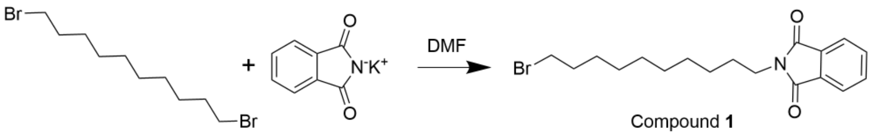

Compound **1**

Under argon atmosphere, 3 eq. of 1,10-dibromodecane (18.2 mL, 80.97 mmol) were added to a suspension of 1 eq. potassium phthalimide (5 g, 27 mmol) in dry DMF (15 mL) and the mixture was stirred at room temperature for 4 h. The solvent was evaporated under vacuum and the residue was dissolved in ethyl acetate. The organic phase was washed with water (2 × 30 mL), saturated aqueous ammonium chloride (1 × 30 mL) and brine (1 × 30 mL). The organic phase was dried over natrium sulphate and the solvent was evaporated in vacuum. After resuspension in heptane, compound 1 was obtained by filtration in a yield of 62 % and directly used for further synthesis.

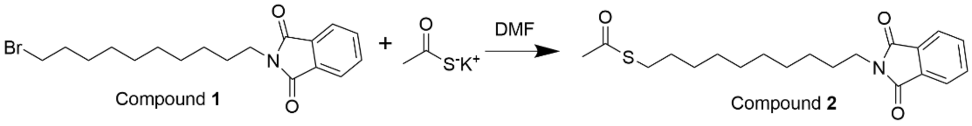

Compound **2**

To the solution of compound 1 (1 eq. 6.14 g, 16.75 mmol) in 10 mL DMF, potassium thioacetate (1.2 eq., 2.3 g, 20.1 mmol) was added and the mixture was stirred under Argon atmosphere at room temperature for 2 h. The solvent was evaporated under vacuum and the residue was dissolved in ethyl acetate. The organic phase was washed with water (2 × 15 mL), saturated ammonium chloride (1 × 15 mL) and brine (1 × 15 mL) and dried over natrium sulphate. The solvent was evaporated under vacuum yielding compound 2 as a brownish viscous liquid in a yield of 93 %.

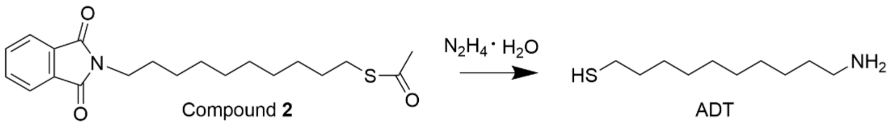

Compound 3 **(ADT)**

Hydrazine hydrate (4 eq., 4 mL, 64.24 mmol) was added to a solution of compound 2 (1 eq., 5.65 g, 15.63 mmol) in methanol (100 mL), and the resulting mixture was stirred under argon atmosphere at 60°C over night. The solvent was removed under vacuum after cooling to room temperature. 20 mL DCM was added to the product. The solution was washed with water (2 × 20 mL) and 1 M sodium hydroxide solution (1 × 20 mL). After drying over natrium sulphate and evaporation of the solvent under vacuum, compound 3 (ADT) was obtained as a white powder in a yield of 50%. ADT was characterized by NMR (Figure S1 and S2) and used directly without further purification.

NMR Characterization

NMR-spectra were recorded on a Bruker Ultrashield 300 MHz NMR-spectrometer (^{1}H NMR: 300 MHz, ^{13}C NMR: 75 MHz). Deuterated chloroform ($CDCl_3$) was used as a solvent (^{1}H NMR: 7.26 ppm, ^{13}C NMR: 77.16 ppm). Spectra were evaluated with Mnova from MESTRELAB RESEARCH.

Electrochemical pore size determination

Following Detsi et al.[1] and Lakshmanan *et al.*[2] the mean pore size of the npAu samples was determined by cyclic voltammetry (CV) in 0.1 M $HClO_4$. CVs were recorded at six equidistant scan rates between 20 and 45 mVs^{-1} as shown in Figure S3(a)) in the double layer regime. The resulting current was plotted *versus* the scan rate and fitted linearly (Figure S3(b)). The slope (*a*)

of the fit gives the total capacitance of the sample. Using the mass of the sample (m, g), the density of gold[3] (ρ = 19.3 g/cm^3), the specific interfacial capacitance[2] (C_{spec} = 40 µF/cm^2) and an empirical dimensionless parameter (k = 3.7 for disordered nanoporous structures[1]), the pore size (d, Eq. S1) can be estimated by

$$d = \frac{mk}{\rho a} * C_{spec} \qquad \text{(S1)}$$

From the same measurement, also the total active surface area (A, Eq. S2) can be determined by

$$A = \frac{a}{C_{spec}} \qquad \text{(S2)}$$

Enzyme activity on polymer carriers

The activity of immobilized LOx on polymer carriers was studied using amine modified (ReliZyme hexamethylamino HA403) and sulfonic acid modified (ReliSorb sulphonic SP400) porous polymethacrylate beads (purchased from Residion S.r.l.). About 250 mg of dry carrier were washed 3 times with KPi buffer in a 2-mL tube. LOx was solubilized in KPi buffer to the desired concentration (~3 mg/mL) and added to the carrier in a volume of 1.0 mL. The beads were incubated for 24 h at 4 °C on a rotator with 40 rpm. Finally, each immobilized preparation was washed at least 5 times with KPi buffer and oxidase activity was determined for approximately 20 mg of the carrier as described in the Experimental section of the manuscript. The amine modified beads showed an immobilization yield of $Y = (99.7 \pm 0.1)\ \%$ with an activity per mass $a_m = (23 \pm 1)\ \mathrm{U/g}$. The sulfonic acid modified beads yielded $Y = (11.0 \pm 0.4)\%$ and $a_m = (3.0 \pm 0.1)\ \mathrm{U/g}$.

Supporting Figures

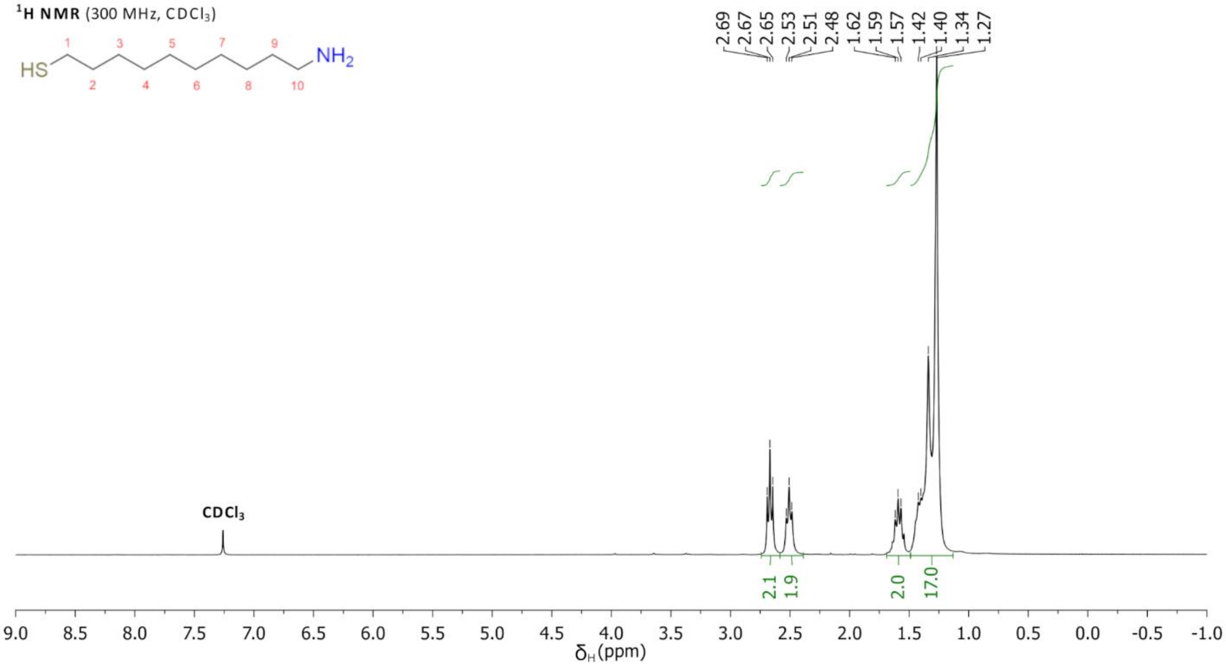

Figure S1. ^{1}H NMR spectrum of ADT (300 MHz, $CDCl_3$): δ_H 2.67 (2H,t), 2.51 (2H,t), 1.59 (2H,quint), 1.40 (2H,quint), 1.2-1.4 (13H, overlapped) ppm. Note: Broad peaks between δ_H 1.2 to 1.4 ppm correspond to the protons of the CH_2 groups on C-3 to C-8, as well as protons of the thiol and amine group, where latter disappeared after D_2O exchange.

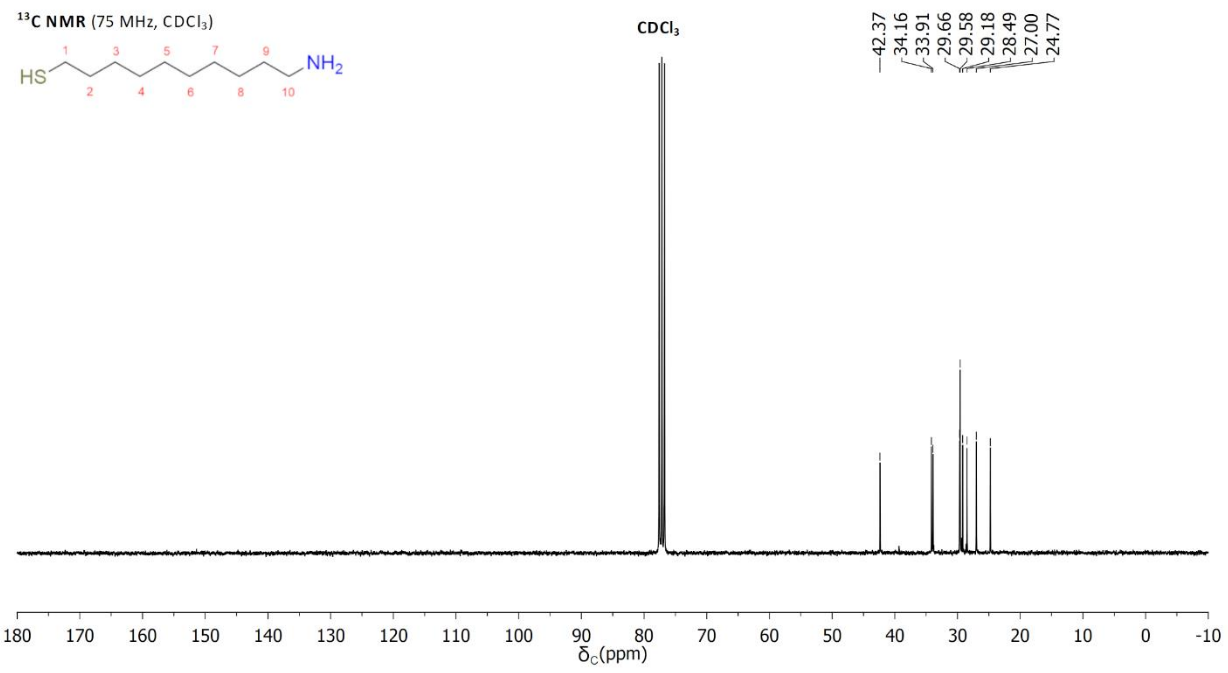

Figure S2. ^{13}C NMR spectrum of ADT (75 MHz, $CDCl_3$): δ_C 42.37, 34.16, 33.91, 29.66, 29.58 (2C), 29.18, 28.49, 27.00, 24.77 ppm.

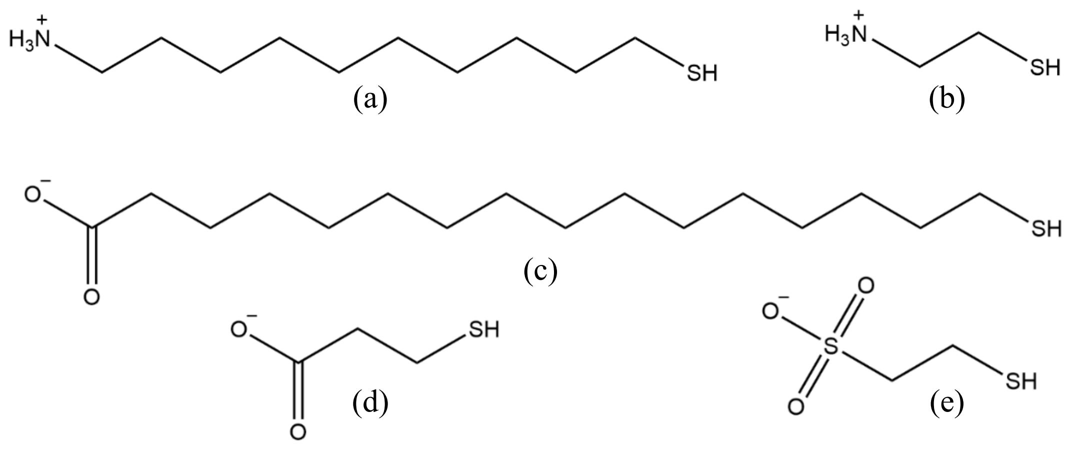

Figure S3. Chemical structure of the SAMs used (dissociated form). (+) and (-) indicate the ionization at pH 7.5. (a) ADT and (b) CYA with an amine as functional group (FG), exhibiting an isoelectric point (IEP) of 9.24 in solution[4]. (c) MHDA and (d) MPA with carboxylic acid FG (IEP 4.3[5]). (e) MESA with sulfonic acid FG (IEP~1.5; calculated from pKa values of the thiol group and the sulfonic acid group).

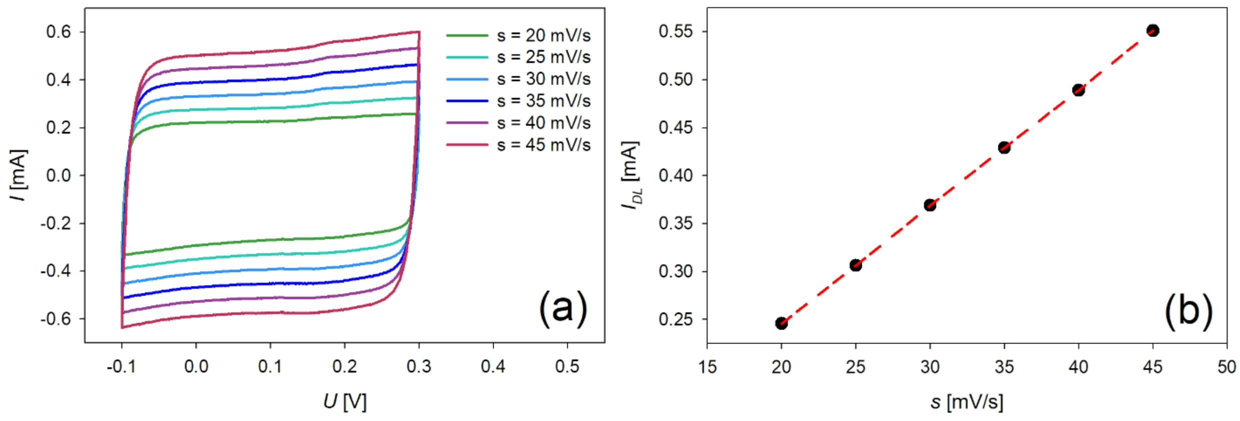

Figure S4. Electrochemical pore size determination. (a) CVs in the double layer regime between -100 and + 300mV in 0.1 M $HClO_4$. Scan rates *s* between 20 and 45 mV/s. (b) Double layer current I_{DL} at 100 mV as function of the scan rate *s* derived from (a). The slope of the linear fit (red) is used to determine the capacitance.

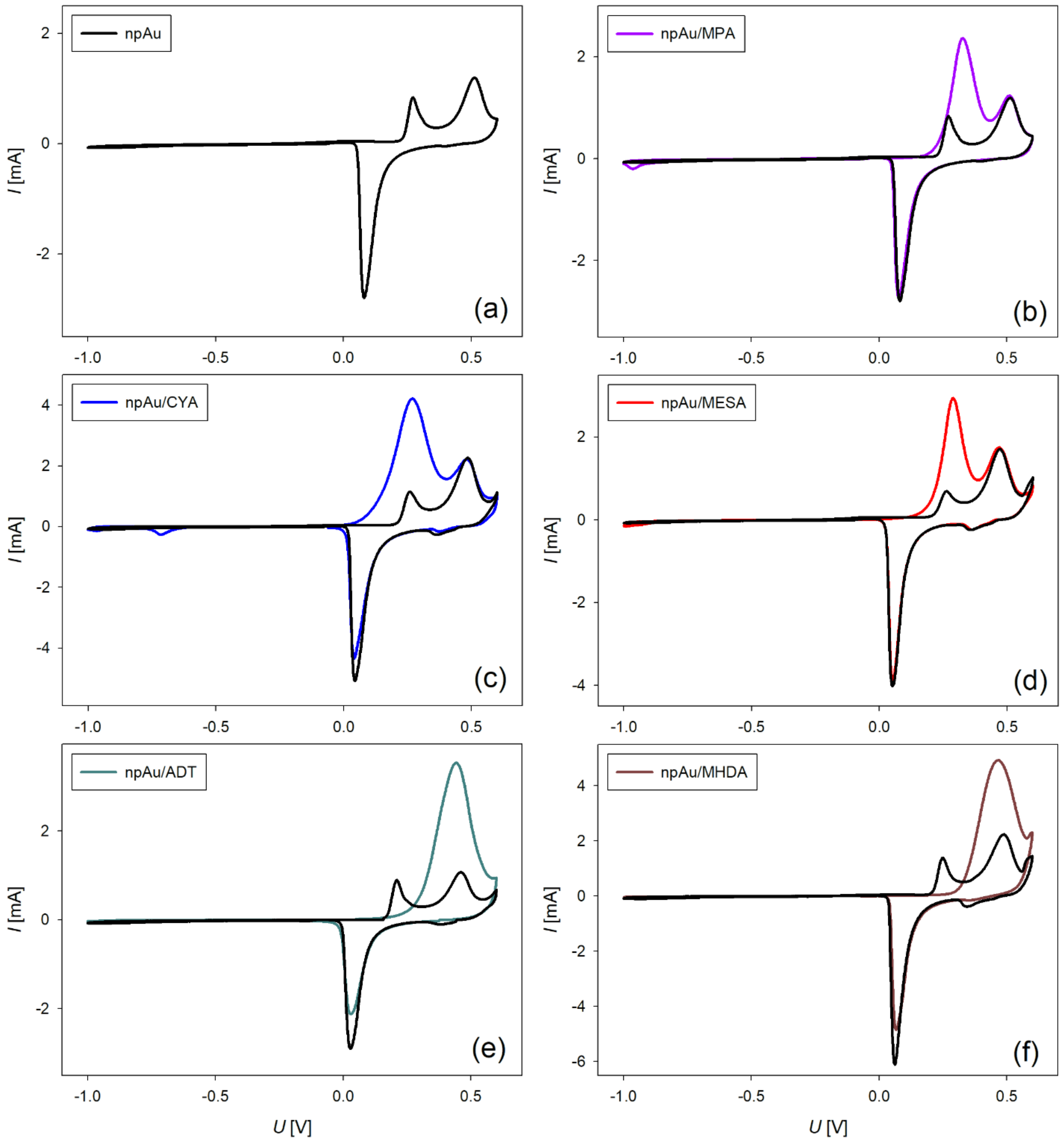

Figure S5. CVs of npAu (black) in 1 M KOH at a scan rate of 1 mV/s. (a) Bare npAu, (b)-(f) show the oxidation peak corresponding to desorption of the different SAMs in the first cycle (coloured). (b) MPA, (c) CYA, (d) MESA, (e) ADT and (f) MHDA.

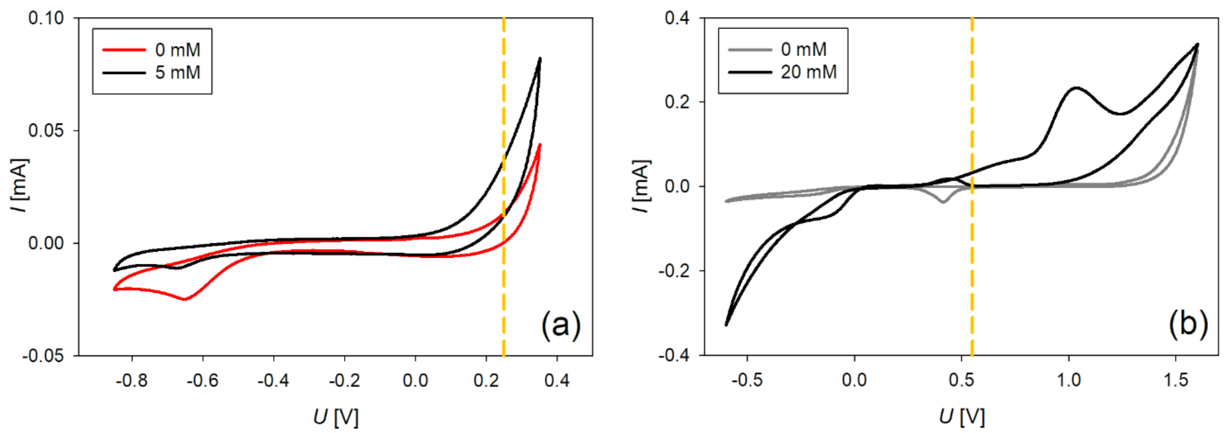

Figure S6. CVs recorded in KPi 50 mM pH 7.5 to determine the potentials used for chronoamperometric (CA) measurements (yellow dashed lines) based on the onset of H_2O_2 oxidation. (a) MESA modified npAu-LOx electrode before (red) and after the addition of 5 mM L-lactate (black). (b) Bare gold wire before (gray) and after the addition of 20 mM H_2O_2 (black). Scan rates were 50 and 5 mV/s, respectively.

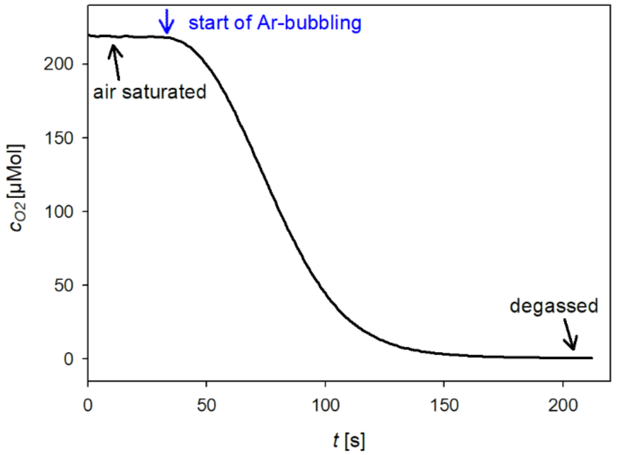

Figure S7. Oxygen sensor calibration. Decrease of the O_2 concentration c_{O2} over time during degassing (start of Ar-bubbling at t = 35 s) and constant stirring at 350 rpm. The measurement starts in air saturated KPi 50 mM, pH 7.5 (first calibration point, 100% O_2) and ends in a fully degassed state (second calibration point, 0% O_2).

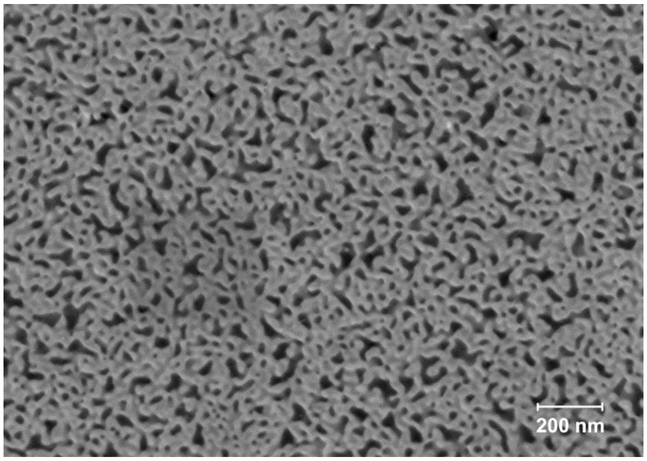

Figure S8. SEM image of npAu. The image was recorded at the cross-section of a freeze-dried and halved electrode to directly analyze the center of the bulk.

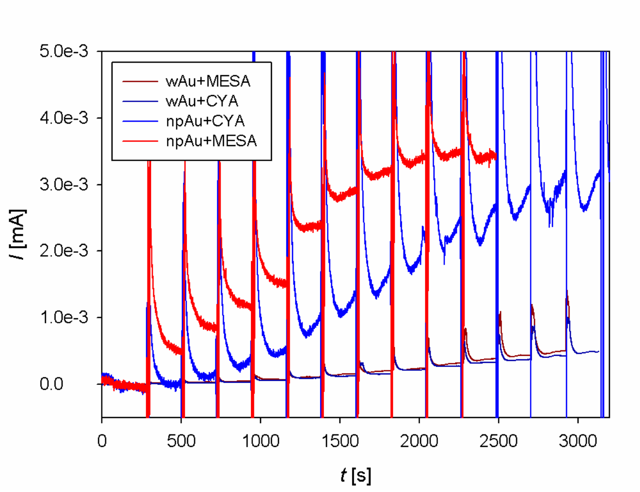

Figure S9. As-recorded CA data for different gold-LOx electrodes in KPi 50 mM showing the current change upon stepwise addition of L-lactate: Au-wires (dark colours) and npAu-electrodes (brighter colours) modified with CYA (blue) and MESA (red). L-lactate concentration was increased stepwise every 220 s starting with 4×50 µM, 4×200 µM and then in 500 µM steps. Current peaks result from electromagnetic stirring for 20 s at 350 rpm during the addition. Applied potentials were +250 mV (npAu) and +550 mV (Au-wire), respectively.

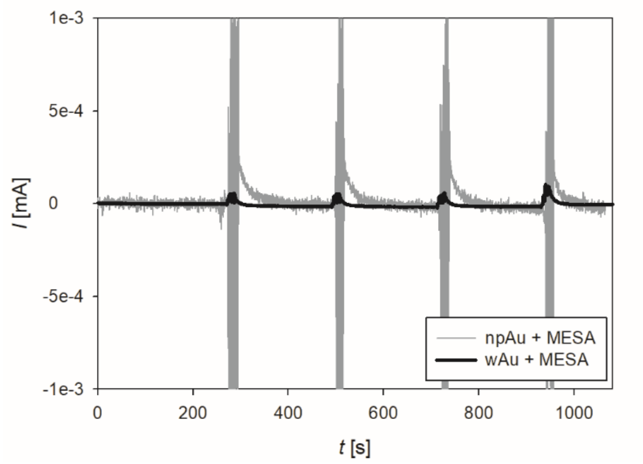

Figure S10. Blank CA measurements of a npAu (gray) and Au-wire (black) modified with MESA (without enzyme) in KPi 50 mM upon stepwise L-lactate addition (4×500 µM, every 220 s) at potentials of +250 and +550 mV, respectively. Current peaks result from electromagnetic stirring for 20 s at 350 rpm during the addition.

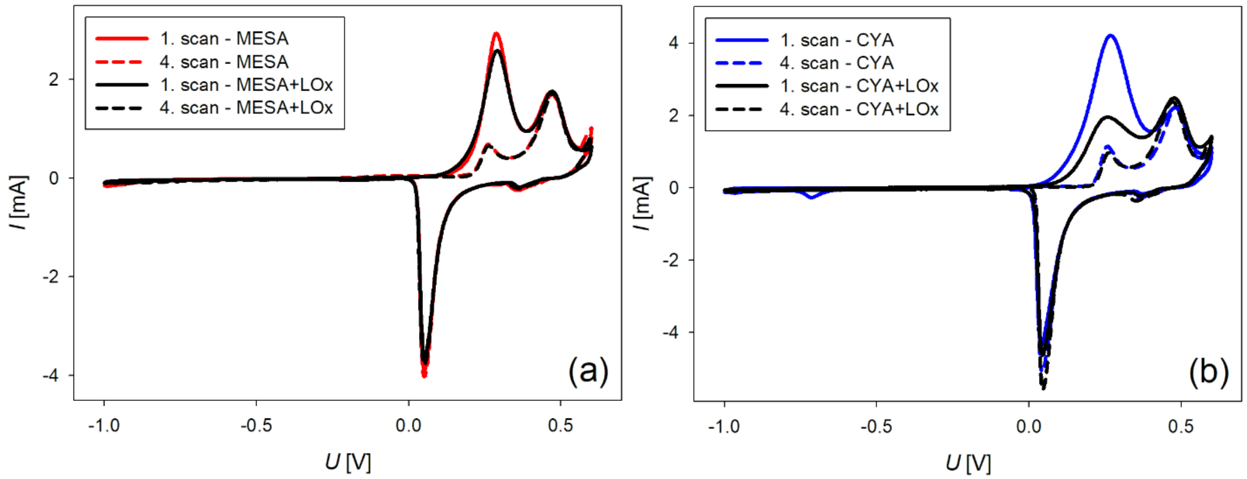

Figure S11. Cyclic voltammograms of npAu in 1M KOH at a scan rate of 2 mV/s to determine the stability of SAMs during CA. (a) MESA and (b) CYA desorption. Coloured curves show the desorption of the SAMs before CA and black curves show the desorption of the SAM together with the immobilized LOx after CA (1st scan). Dashed lines show the corresponding cleaned bare npAu sample (4th scan).

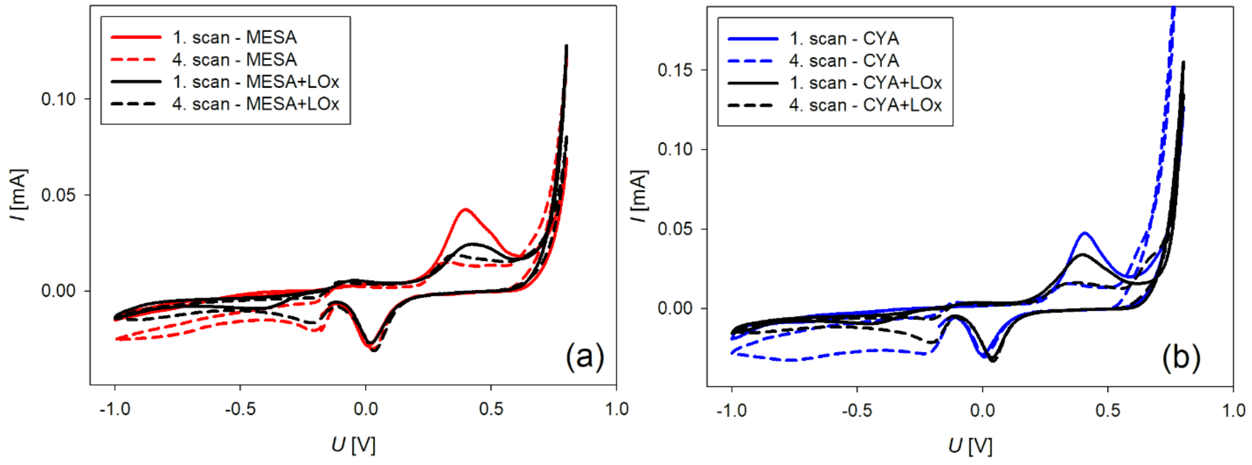

Figure S12. CVs of Au-wires in 1M KOH at a scan rate of 50 mV/s to determine the stability of SAMs during CA. (a) MESA and (b) CYA desorption. Coloured curves show the desorption of the SAMs before CA and black curves show the desorption of the SAM together with the immobilized LOx after CA (1st scan). Dashed lines show the corresponding cleaned bare Au-wire (4th scan).

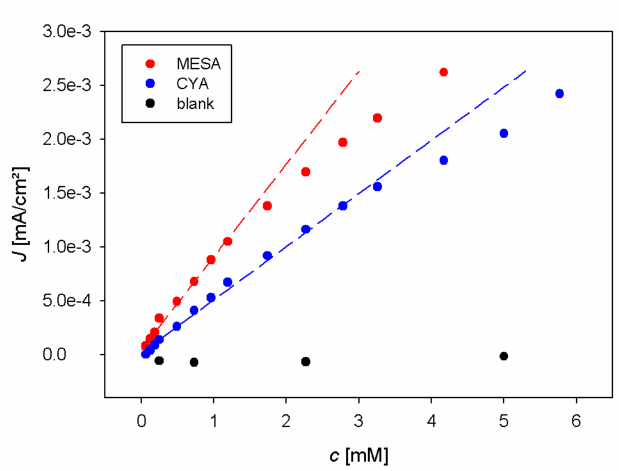

Figure S13. Mean current density *j* over L-lactate concentration *c* as determined from CA for Au-wire-LOx electrodes for a MESA (red) and a CYA (blue) modified sample in KPi 50 mM at a potential of +550 mV. Dashed lines represent fits of the linear part of the response. A blank measurement (black) was performed using Au-wire-MESA without enzyme.

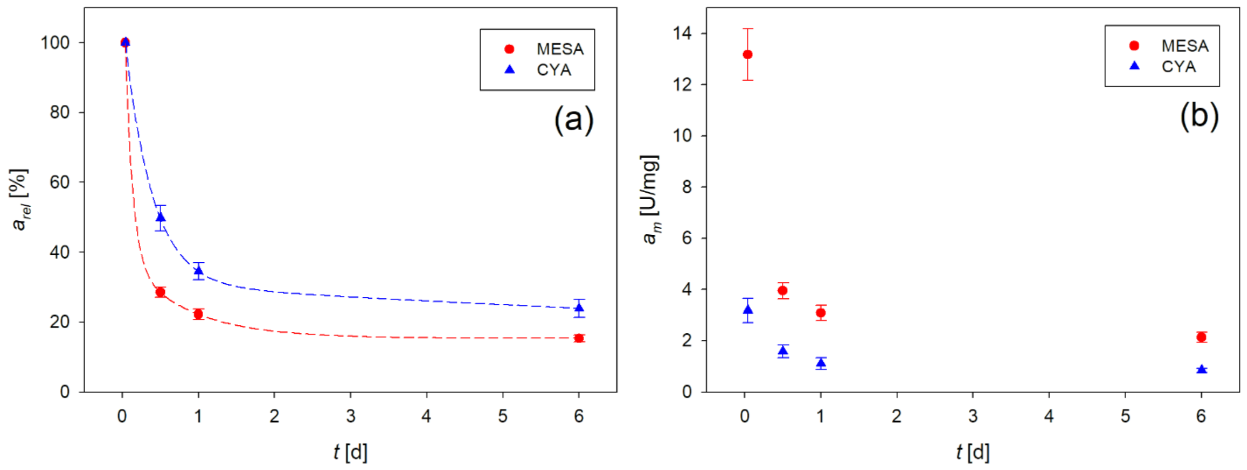

Figure S14. Elution of LOx from npAu electrodes with a CYA (blue) and MESA (red) SAM over a time t of 6 days using the same set of samples as in Table 1 ($N = 3$). The first data point ($t = 0$ d) corresponds to the activity right after the immobilization process. (a) Relative decrease of the activity (a_{rel} in %) with respect to the activity right after immobilization for each sample. Dashed lines represent spline fits as a guide for the eye. (b) Absolute change of activity per mass (a_m in U/g) over time.

References

(1) Detsi, E.; Jong, E.; Zinchenko, A.; Vuković, Z.; Vuković, I.; Punzhin, S.; Loos, K.; Brinke, G.; Raedt, H. A.; Onck, P. R.; Hosson, J. T. M. On the Specific Surface Area of Nanoporous Materials. *Acta Mater.* **2011**, *59*, 7488–7497.

(2) Lakshmanan, C.; Viswanath, R. N.; Polaki; Rajaraman, R. Determination of Surface Area of Nanoporous Metals: Insights from Double Layer Charging. In *AIP Conference Proceedings*; Bhattacharyya, D., Chitra R, Sahoo, N. K., Eds.; AIP Publishing: Melville, NY, 2015; p 140033.

(3) Habashi, F. Gold, Physical and Chemical Properties. In *Encyclopedia of Metalloproteins*; Kretsinger, R. H., Uversky, V. N., Permyakov, E. A., Eds.; Springer New York: New York, NY, 2013; pp 932–933.

(4) Ma, Q.; Fang, X.; Zhang, J.; Zhu, L.; Rao, X.; Lu, Q.; Sun, Z.; Yu, H.; Zhang, Q. Discrimination of Cysteamine from Mercapto Amino Acids through Isoelectric Point-Mediated Surface Ligand Exchange of β-Cyclodextrin-Modified Gold Nanoparticles. *J. Mater. Chem. B* **2020**, *8*, 4039–4045.

(5) Schweiss, R.; Welzel, P.; Knoll, W.; Werner, C. Assembly Modulates Dissociation: Electrokinetic Experiments Reveal Peculiarities of the Charge Formation at Monolayer Films. *Chem. Commun.* **2005**, No. 2, 256–258.
